# The MAPK and AMPK signalings: interplay and implication in targeted cancer therapy

**DOI:** 10.1186/s13045-020-00949-4

**Published:** 2020-08-17

**Authors:** Jimin Yuan, Xiaoduo Dong, Jiajun Yap, Jiancheng Hu

**Affiliations:** 1 Department of Urology, Shenzhen People’s Hospital (The Second Clinical Medical College, Jinan University; The First Affiliated Hospital, Southern University of Science and Technology), Shenzhen, 518020 Guangdong China; 2 Geriatric Department, Shenzhen People’s Hospital (The Second Clinical Medical College, Jinan University; The First Affiliated Hospital, Southern University of Science and Technology), Shenzhen, 518020 Guangdong China; 3 Shenzhen People’s Hospital, 1017 Dongmen North Road, Shenzhen, 518020 China; 4 Cancer and Stem Cell Program, Duke-NUS Medical School, 8 College Road, Singapore, 169857 Singapore; 5 Division of Cellular and Molecular Research, National Cancer Centre Singapore, 11 Hospital Drive, Singapore, 169610 Singapore

**Keywords:** Ras/RAF/MEK/ERK signaling, AMPK signaling, Interplay, Tumorigenesis, Cellular metabolism, RAF/MEK/ERK inhibitors, AMPK inhibitors, AMPK activators, Autophagy, Targeted therapy

## Abstract

Cancer is characterized as a complex disease caused by coordinated alterations of multiple signaling pathways. The Ras/RAF/MEK/ERK (MAPK) signaling is one of the best-defined pathways in cancer biology, and its hyperactivation is responsible for over 40% human cancer cases. To drive carcinogenesis, this signaling promotes cellular overgrowth by turning on proliferative genes, and simultaneously enables cells to overcome metabolic stress by inhibiting AMPK signaling, a key singular node of cellular metabolism. Recent studies have shown that AMPK signaling can also reversibly regulate hyperactive MAPK signaling in cancer cells by phosphorylating its key components, RAF/KSR family kinases, which affects not only carcinogenesis but also the outcomes of targeted cancer therapies against the MAPK signaling. In this review, we will summarize the current proceedings of how MAPK-AMPK signalings interplay with each other in cancer biology, as well as its implications in clinic cancer treatment with MAPK inhibition and AMPK modulators, and discuss the exploitation of combinatory therapies targeting both MAPK and AMPK as a novel therapeutic intervention.

## Introduction

The Ras/RAF/MEK/ERK (MAPK) signaling is a fundamental pathway in cell biology, and its alteration causes human cancers or developmental disorders. Given its crucial roles in physiology and pathology, this pathway has been extensively studied for over two decades. Unfortunately, the regulation of MAPK signaling remains ambiguous till now by virtue of its intrinsic complexity and diverse crosstalks with other signalings. Here, we focus on the complicated interplays between the MAPK and the AMPK signalings in cellular carcinogenesis and their implications in current targeted cancer therapies. We hope this review would provide a conceptual framework for developing more effective therapeutic approaches against hyperactive MAPK signaling-driven cancers.

## The Ras/RAF/MEK/ERK (MAPK) signaling and its aberrant activation in cancers

### The Ras/RAF/MEK/ERK (MAPK) signaling

The Ras/RAF/MEK/ERK (MAPK, mitogen-activated protein kinase) signaling is a central pathway that regulates cellular proliferation, differentiation, and survival. This signaling pathway was discovered in the 1970s**–**1980s, when Ras small GTPases were identified as first oncogenes from sarcoma viruses [1–6]. Later, studies on viral oncogenes had also led to the discovery of a N-terminal truncated version of RAF Ser/Thr kinase (RAF1 or CRAF) [1–5]. In contrast, the other two components of this signaling pathway, MEK (mitogen-activated protein kinase kinase) and ERK (mitogen-activated protein kinase) were identified as cytoplasmic protein kinases activated by mitogens in the 1990s [7–11]. Following these discoveries, RAF was identified as the upstream kinase of MEK in 1992 and the first direct effector of Ras in 1993 [12, 13], resulting in the delineation of the whole MAPK signaling pathway, which is considered as a milestone in our understanding of how cell senses external stimuli.

The first component of MAPK signaling, Ras small GTPases, have three gene isoforms: H-ras, K-ras, and N-ras, that encode four proteins with splicing isoforms of K-ras giving rise to K-ras4A and K-ras4B. Although all Ras proteins possess highly homologous sequences, they have quite different activities, tissue expression patterns, and effector preferences, which lead to their differential physiological and pathological functions [14–17].

The downstream of Ras small GTPases is the RAF/MEK/ERK kinase cascade [18]. The first kinases in this cascade, RAF/KSR (kinase suppressor of Ras) family kinases, include three RAF isoforms, i.e., CRAF, BRAF, and ARAF, and two close pseudokinases, i.e., KSR1 and KSR2. All RAF isoforms have highly homologous sequences and similar structures with three conserved regions: conserved region 1 (CR1) contains RAS-binding domain (RBD) and a Cys-rich domain [19, 20]; conserved region 2 (CR2) is characterized by a Ser/Thr-rich sequence; conserved region 3 (CR3) comprises of a putative kinase domain with a N-terminal acidic motif (NTA) [21–23] and a C-terminal regulatory tail [24–26]. Nevertheless, RAF isoforms have variable kinase activities with an order as BRAF>CRAF>ARAF likely by virtue of their distinct NTA motifs and APE motifs that contribute to the dimerization-driven transactivation of RAFs [27–30]. In contrast to RAF isoforms, KSR proteins replace the RBD at the N-terminus with a coiled-coil fused sterile α-motif and Pro-rich stretch that are responsible for recruiting proteins to the plasma membrane upon stimulation, and lack the catalytic lysine in VAIK motif of kinase domain which impairs their catalytic activity [31, 32]. Given their associations with MEK and ERK as well as low kinase activity, KSR proteins have been thought as scaffold proteins in a long term. However, recent studies have indicated that KSR proteins can also function as allosteric activators to stimulate the catalytic activity of RAF proteins through dimerization [27, 32–37]. The side-to-side dimerization of RAF/KSR family kinases is critical not only for their activation but also for their catalytic activity towards downstream kinases [25, 38–42]. MEKs (MEK1 and MEK2) are the second kinases of the RAF/MEK/ERK kinase cascade, which have both redundant and non-redundant functions [43, 44]. These two dual-specific kinases comprise a short regulatory N-terminus and a canonic kinase domain. The N-terminal regulatory region of MEK1/2 contains a docking site for substrate ERKs, a nuclear export sequence that controls the cytoplasmic-nuclear shuttling of proteins, and a negative regulatory sequence that forms a helix and locks kinase in an inactive conformation [11, 43, 44]. Further, through its kinase domain, MEK1/2 forms a face-to-face heterodimer with RAF/KSR, or a homodimer/heterodimer with itself, which is indispensable for its activation stimulated by RAF and for its activity towards ERKs [28, 45, 46]. Like MEKs, the terminal kinases of MAPK signaling, ERKs, also include two highly homologous members, ERK1 and ERK2, which have a central kinase domain flanked by short N- and C-terminal tails. These two isoforms also have redundant functions albeit different expression patterns [7–10]. However, unlike RAFs and MEKs that have very limited substrates, ERKs recognize and phosphorylate numerous substrates that include transcription factors, protein kinases and phosphatases, and other functional proteins [47–51].

It should be noted that active Ras also turns on other signaling pathways such as PI3K/AKT/mTORC, which regulate different cellular functions [52]. In this review, we focus only on the MAPK signaling given its dominant role in cancer biology.

### Hyperactive Ras/RAF/MEK/ERK (MAPK) signaling in cancers

The MAPK signaling plays a crucial role in cell biology and is tightly regulated in normal cells. Upon engagement of receptor tyrosine kinases (RTKs) or other stimulations, Ras small GTPases are activated by GTP/GDP exchange factors (GEFs), which in turn recruit RAF/MEK complexes to the plasma membrane and trigger the RAF/MEK/ERK kinase cascade through facilitating RAF/RAF (or KSR), RAF/MEK, and MEK/MEK interactions as well as subsequent phosphorylations [53]. Active ERKs are further translocated into the nuclei or stay in the cytoplasm, where they phosphorylate a number of substrates that regulate cell functions [49–51, 54, 55]. On the other hand, active MAPK signaling also turns on some negative feedback loops, which help cells return to quiescent status [56–58]. An aberrant activation of MAPK signaling frequently induces human cancers or developmental disorders, though an extremely high MAPK signaling may induce cell death or senescence under some conditions [59–63].

Hyperactive MAPK signaling exists in over 85% of cancers, which is caused directly by genetic alterations of its upstream activators or components, including RTKs, Ras, and BRAF, or indirectly by those independent of Ras or RAF [64–66], and significantly promotes disease progression [67]. Since genetic alterations of RTKs in cancers have been extensively reviewed in recent years [68–73], here we focus on oncogenic mutations of Ras and BRAF. As a small GTPase, Ras cycles between active GTP-bound status and inactive GDP-bound status, which is regulated by GEFs and GTPase-activating proteins (GAPs). Oncogenic Ras mutations can be mainly classified into two groups: (1) mutations on glycine 12 or 13 (G12/13) that impair GAP associations and (2) mutations on glutamine 61 (Q61) that diminish the intrinsic GTPase activity of Ras [74], both of which lead to an extended half-life of GTP-loaded Ras. Oncogenic Ras mutations have both isoform and cancer-type preferences. K-ras is mostly mutated in all cancers (85%), followed by N-ras (12%) and H-ras (3%), and its mutations prevail in pancreatic cancers, while those of N-ras in myeloma and melanomas, and H-ras in adrenal gland cancers [75, 76]. This phenomenon may reflect underlying fundamental signaling landscapes, and RAS mutants interplay with these landscapes. As the downstream effector of Ras, RAF is another dominant target of oncogenic mutations in the MAPK signaling pathway. Similarly, RAF mutations have isoform preference in cancers as Ras mutations with BRAF >> CRAF > ARAF, which may arise from their different basal activities. Overall, a single point mutation that converts Val 600 into Glu in the activation loop of BRAF accounts for > 90% cases [77]. Although BRAF (V600E) exists only in ~ 7% of all cancers, it is highly prevalent in some tissue-specific cancers such as melanoma (50~60%), thyroid cancer (40~50%), and histiocytosis (~50%) [78–81], albeit the underlying molecular mechanism(s) remains unknown. In contrast to Ras and RAF, MEK and ERK have rare mutations in cancers though their mutations have been shown to be responsible for some RAF inhibitor (RAFi)-resistant cases in current cancer therapies [82–85].

### Targeting the Ras/RAF/MEK/ERK (MAPK) signaling pathway for cancer therapy: promising but challenging

**Fig. 1:**
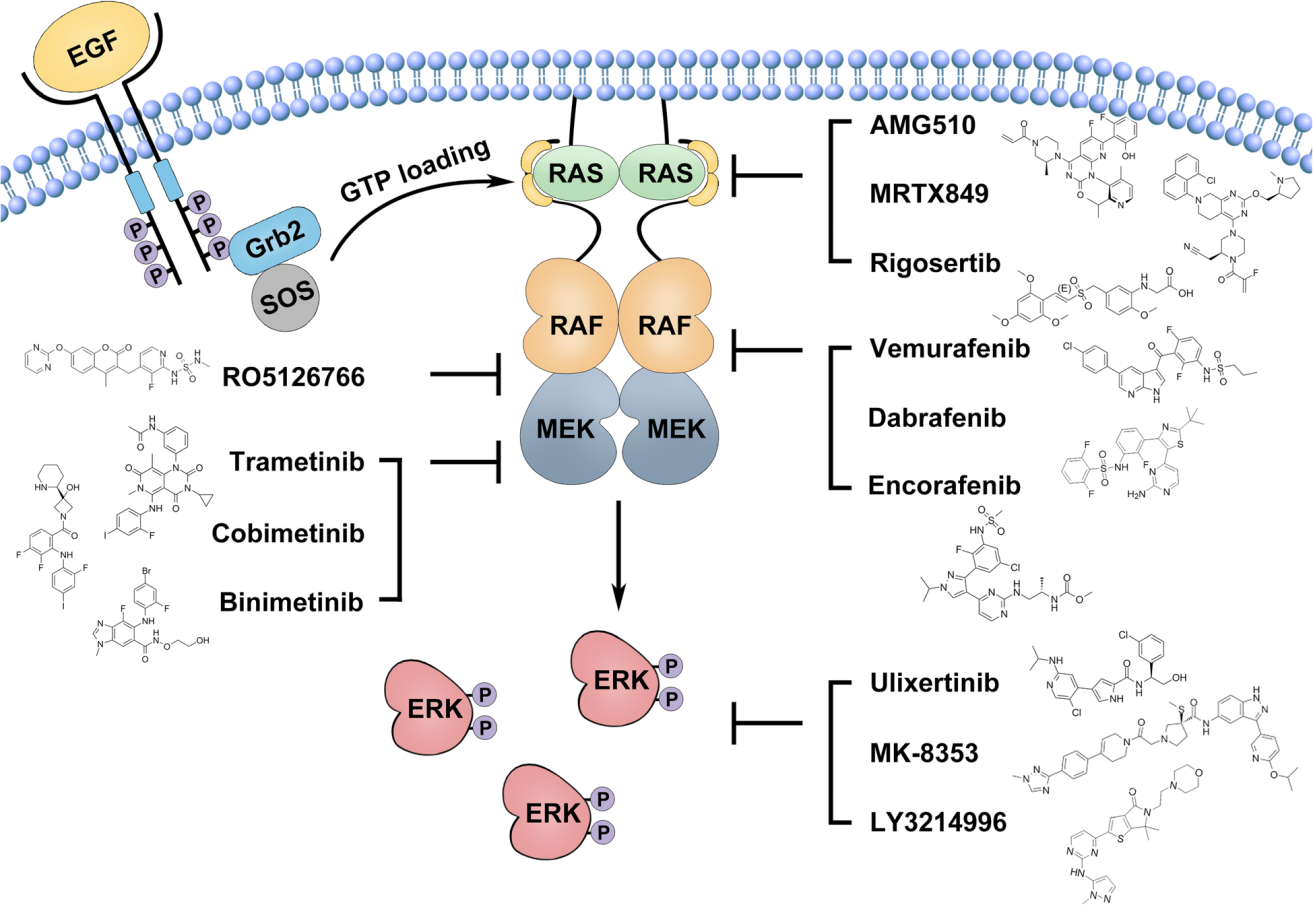
Target hyperactive Ras/RAF/MEK/ERK (MAPK) signaling for cancer therapy. The Ras/RAF/MEK/ERK (MAPK) signaling functions downstream of receptor tyrosine kinases (RTKs). Upon engagement by their ligands, RTKs activates guanine exchange factors, Sos proteins, which load GTP to Ras GTPases. Then, GTP-bound Ras GTPases recruit RAF/MEK heterodimers in cytosol to plasma membrane where they form transient tetramers through the side-to-side dimerization of RAFs. The RAF dimerization not only turns on RAFs but also loosens RAF/MEK heterodimerization and facilitates MEK homodimerization on RAF dimer surface, which leads to the activation of MEKs by RAFs. Once MEKs are activated, they phosphorylate ERKs, and then active ERKs phosphorylate a number of downstream effectors. In cancer cells, hyperactive Ras/RAF/MEK/ERK (MAPK) signaling arising from genetic mutations of Ras GTPases and BRAF can be targeted by small molecular inhibitors of Ras G12C, BRAF(V600E), MEK, and ERK

Given their high prevalence in cancers, great efforts have been made to develop specific inhibitors against oncogenic Ras and RAF mutants in the last decades. These inhibitors that have been approved for clinic treatment of Ras/RAF-mutated cancers or under clinical trials are listed in Table 1. However, none of these inhibitors can effectively target the large portion of Ras mutants in cancers. Since having no attractive docking sites suitable for designing high-affinity and selective small molecule inhibitors, Ras mutants have been thought as “undruggable” cancer drivers in a long term. Until recently, a group of covalent small inhibitors that are docked into a previously unknown pocket of GDP-bound Ras and are linked to the adventive cysteine of Ras(G12C) have been developed and achieved encouraging outcomes for treating Ras(G12C)-driven cancers as a single agent in clinical trials [86–91] (Fig. 1). To further enhance their efficacy, these Ras(G12C) inhibitors are also undergoing clinical evaluation when combined with SHP2 (Src homology region 2 domain-containing phosphatase-2) inhibitors that block the pathway reactivation caused by the relief of negative feedback loops [92, 93] (Clinical Trial: NCT04330664). In addition, these inhibitors have also been further developed into Ras(G12C) degraders by conjugating with ligands of ubiquitin E3 ligases, which effectively deplete Ras mutant proteins in cancer cells [94, 95] though their efficacy in vivo remains unknown. Unlike Ras(G12C), the majority of Ras mutants remain “undruggable” at present [96].

**Table 1:** Summary of small molecule inhibitors approved and under clinical trials for treating Ras/RAF-mutated cancers

Target	Compound	Development stages	Description
KRas G12C	AMG-510	Phase III, NCT04303780	Phase I results showed 54% ORR of non-small cell lung cancer (NSCLC) harboring KRas G12C.
	MRTX849	Phase I/II, NCT03785249 Phase I/II, NCT04330664	Evaluation of clinical activity of MRTX849 alone and combined with TNO155 (SHP2 inhibitor) in KRas G12C mutated cancers.
	JNJ-74699157	Phase I, NCT04006301	Safety and PK of JNJ-74699157.
Ras	Rigosertib	Phase I/II, NCT04263090	Evaluation of safety and clinical efficacy of Rigosertib plus Nivolumab (PD-1 Ab) in KRas mutated NSCLC.
BRAF	Vemurafenib	Approved	Late-stage or unresectable melanoma expressing BRAF V600E in 2011. Erdheim-Chester disease (ECD) with BRAF V600E mutation in 2017.
	Dabrafenib	Approved	Late-stage or unresectable melanoma expressing BRAF V600E in 2013. Combination with trametinib for the treatment of unresectable or metastatic melanoma with BRAF V600E/K in 2014. Combination with trametinib for the treatment of metastatic NSCLC with BRAF V600E in 2017. Combination with trametinib for the adjuvant treatment of melanoma with BRAF V600E/K in 2018. Combination with trametinib for the treatment of anaplastic thyroid cancer (ATC) that cannot be removed by surgery or has spread to other parts of the body with BRAF V600E in 2018.
	Encorafenib	Approved	Combination with binimetinib for the treatment of patients with unresectable or metastatic melanoma with BRAF V600E/K in 2018. Combination with cetuximab (EGFR Ab) for the treatment of metastatic colorectal cancer with BRAF V600E in 2020.
	PLX8394	Phase I/II, NCT02428712	PLX8394 with cobicistat (CYP3A inhibitor) was well tolerated and showed promising activity in BRAF-mutated refractory cancers.
	BGB283	Phase I, NCT02610361 Phase I/II, NCT03905148	Evaluation of safety and PK of BGB-283 alone and combination with mirdametinib.
	TAK-580	Phase I, NCT02327169 Phase I, NCT03429803	TAK-580 is the inhibitor of BRAF V600E and dimers. Treatment in pediatric low-grade glioma.
	CCT3833	Phase I, NCT02437227	CCT3833 is a pan-RAF inhibitor of mutant BRAF, CRAF and SRC kinases.
RAF/MEK	RO5126766	Phase I, NCT00773526 Phase I, NCT03681483 Phase I, NCT03875820 Phase I, NCT02407509	RO5126766 is a dual inhibitor for both RAF and MEK. Treatment of advanced KRas-mutant lung adenocarcinomas. Evaluation of safety and PK of RO5126766 with VS-6063 (FAK inhibitor) or everolimus (mTOR inhibitor). RO5126766 showed activity across Ras- and RAF-mutated malignancies, with significant response in lung and gynecological cancers.
MEK1/2	Trametinib	Approved	A single-agent oral treatment for unresectable or metastatic melanoma with BRAF V600E/K in 2013. Combination with dabrafenib for the treatment of unresectable or metastatic melanoma with BRAF V600E/K in 2014. Combination with dabrafenib for the treatment of metastatic NSCLC with BRAF V600E in 2017. Combination with dabrafenib for the adjuvant treatment of melanoma with BRAF V600E/K in 2018. Combination with dabrafenib for the treatment of ATC that cannot be removed by surgery or has spread to other parts of the body with BRAF V600E in 2018.
	Cobimetinib	Approved Phase I/II, NCT03989115	In combination with vemurafenib to treat advanced melanoma with BRAF V600E/K in 2015. Dose-escalation of combination of RMC-4630 (SHP2 inhibitor) and cobimetinib.
	Binimetinib	Approved	Combination with encorafenib for the treatment of patients with unresectable or metastatic melanoma with BRAF V600E/K in 2018.
	Selumetinib	Approved	Selumetinib was approved for neurofibromatosis type 1 with symptomatic, inoperable plexiform neurofibromas according to NCT01362803
	Mirdametinib	Phase II, NCT03962543 Phase II, NCT02022982 Phase I/II, NCT03905148	Evaluation of mirdametinib in the treatment of symptomatic inoperable neurofibromatosis type-1 (NF1)-associated plexiform neurofibromas (PNs). Combination of mirdametinib with palbociclib in the treatment of KRas mutant non-small cell lung cancer (NSCLC). Evaluation of safety and PK of BGB-283 alone and combination with mirdametinib.
	SHR-7390	Phase I, NCT02968485	Evaluation of safety and PK of SHR-7390.
	CS-3006	Phase I, NCT03516123 Phase I, NCT03736850	Evaluation of safety and PK of CS-3006.
ERK1/2	Ulixertinib	Phase I/II, NCT01781429 Phase I, NCT04145297 Phase II, NCT03698994 Phase I, NCT03454035	Responses to ulixertinib in NRas, BRAF V600 and non-V600 BRAF mutant cancers. Evaluation of ulixertinib alone or combined with hydroxychloroquine, palbociclib (CDK4/6 inhibitor) in MAPK mutated cancers.
	MK-8353	Phase I, NCT01358331 Phase I, NCT03745989 Phase I, NCT02972034	MK-8353 was optimized from SCH772984 for better pharmacokinetics, and exhibited inhibition of BRAF V600 mutant cancers. Evaluation of combination of MK-8353 with selumetinib or pembrolizumab (PD-1 Ab) in advanced malignancies.
	LY3214996	Phase I, NCT04081259 Phase I, NCT04391595 Phase I, NCT02857270 Phase II, NCT04386057	Evaluation of treatment of MK-8353 alone or combined with abemaciclib (CDK4/6 inhibitor), Hydroxychloroquine in advanced malignancies.
	ASTX029	Phase I/II, NCT03520075	Evaluation of safety and PK of ASTX029.
	ATG-017	Phase I, NCT04305249	Evaluation of safety and PK of ATG-017.
	KO-947	Phase I, NCT03051035	Evaluation of safety and PK of KO-947.

It has been shown that Ras activates downstream effectors through direct interactions. Therefore, disrupting Ras/effector interactions might be an alternative approach that can effectively block cancer growth driven by Ras mutations. Such a type of small molecule blockers include rigosertib, sulindac, and MCP110, and among which, the therapeutic efficacy of rigosertib combined with nivolumab for Ras-mutated cancers is being determined by phase I/II clinical trials currently [97] (Clinical Trial: NCT04263090). However, it has to be noted that these inhibitors impair the MAPK signaling in both Ras-mutated cancers and normal tissues and thereby their therapeutic index may not be high.

Genetic studies have revealed that the ablation of the RAF/MEK/ERK kinase cascade but not other effector pathways is a most efficient approach to inhibit the growth of Ras-mutated cancers [98], which leads to extensive developments of specific inhibitors against this kinase cascade for treating Ras-mutated cancers. Moreover, these inhibitors should be also effective for treating RAF-mutated cancers. Indeed, a number of RAF/MEK/ERK inhibitors have been developed and applied to clinical trials for treating Ras/RAF-mutated cancers [67, 99–107]. At present, three RAF inhibitors and three MEK inhibitors have been approved to treat late-stage BRAF(V600E)-harboring cancers as a single agent or in combination with other chemotherapeutics and exhibited excellent efficacies [101, 108–116] (Fig. 1). However, Ras-mutated cancers possess intrinsic resistance to both RAF and MEK inhibitors [98], and even BRAF (V600E)-harboring cancers develop acquired resistance after 6–10 months treatment [111, 117]. Mechanistic studies have shown that active Ras facilitates the RAF dimerization on plasma membrane, which leads to both intrinsic and acquired resistance to RAF inhibitors [118–120]. To overcome the drug resistance arising from enhanced RAF dimerization, the second-generation RAF inhibitors such as PLX8394, BGB283, TAK-580, and CCT3833 have been developed and are undergoing clinical evaluations (Clinical Trials: NCT02428712, NCT02610361, NCT03905148, NCT02327169, NCT02437227). These novel RAF inhibitors reduce the RAF dimerization-driven resistance through distinct mechanisms: (1) PLX8394 and BGB283 impair RAF dimerization upon loading on RAF proteins [121–123]; (2) TAK-580 binds to and inhibits both protomers in RAF dimers [124]; (3) CCT3833 inhibits both RAF and upstream kinases of Ras and thereby prevents the activation of Ras by the relief of negative feedback loops [125, 126]. Besides these second-generation RAF inhibitors, a unique RAF/MEK dual inhibitor, RO5126766, has been developed and exhibited a strong potential against both Ras- and RAF-mutated cancers in phase I clinical trials [127–130]. This allosteric inhibitor docks on MEK and prevents the release of MEK from RAF as well as the subsequent phosphorylation of MEK by RAF [128], which gives it much more advantages than all other known RAF inhibitors according to the regulatory mechanism of the RAF/MEK/ERK kinase cascade [46]. As to small molecule inhibitors that target the terminal kinase, ERK, although a number of them have been developed and are undergoing clinical trials [131, 132], their therapeutic values for treating Ras/RAF-mutated cancers remain unknown. Like MEK inhibitors, these ERK inhibitors may not achieve a good therapeutic index as single agents by virtue of their inhibitory role in both malignant and normal tissues. However, they may contribute to anti-Ras/RAF cancer therapy as synergetic agents combined with Ras/RAF inhibitors.

Overall, targeting hyperactive MAPK signaling has achieved exciting outcomes for treating Ras/RAF-mutated cancers. However, although some effective small molecule inhibitors have been developed and applied to clinical treatment, drug resistance and side effects remain remarkable challenges and there is still a long way to develop a long-effective approach with manageable side effects for treating Ras/RAF-mutated cancers.

Although hyperactive MAPK signaling has a dominant role in cancer biology, it is fine-tuned by other signalings such as PI3K/AKT/mTORC and AMPK during disease progression [133]. These signaling interplays have important impacts on both cancer progression and clinical treatment based on MAPK inhibition. In this review, we will focus on the crosstalk between MAPK and AMPK signalings.

## AMPK signaling and its roles in cancer biology

### AMPK signaling and cellular metabolism

**Fig. 2:**
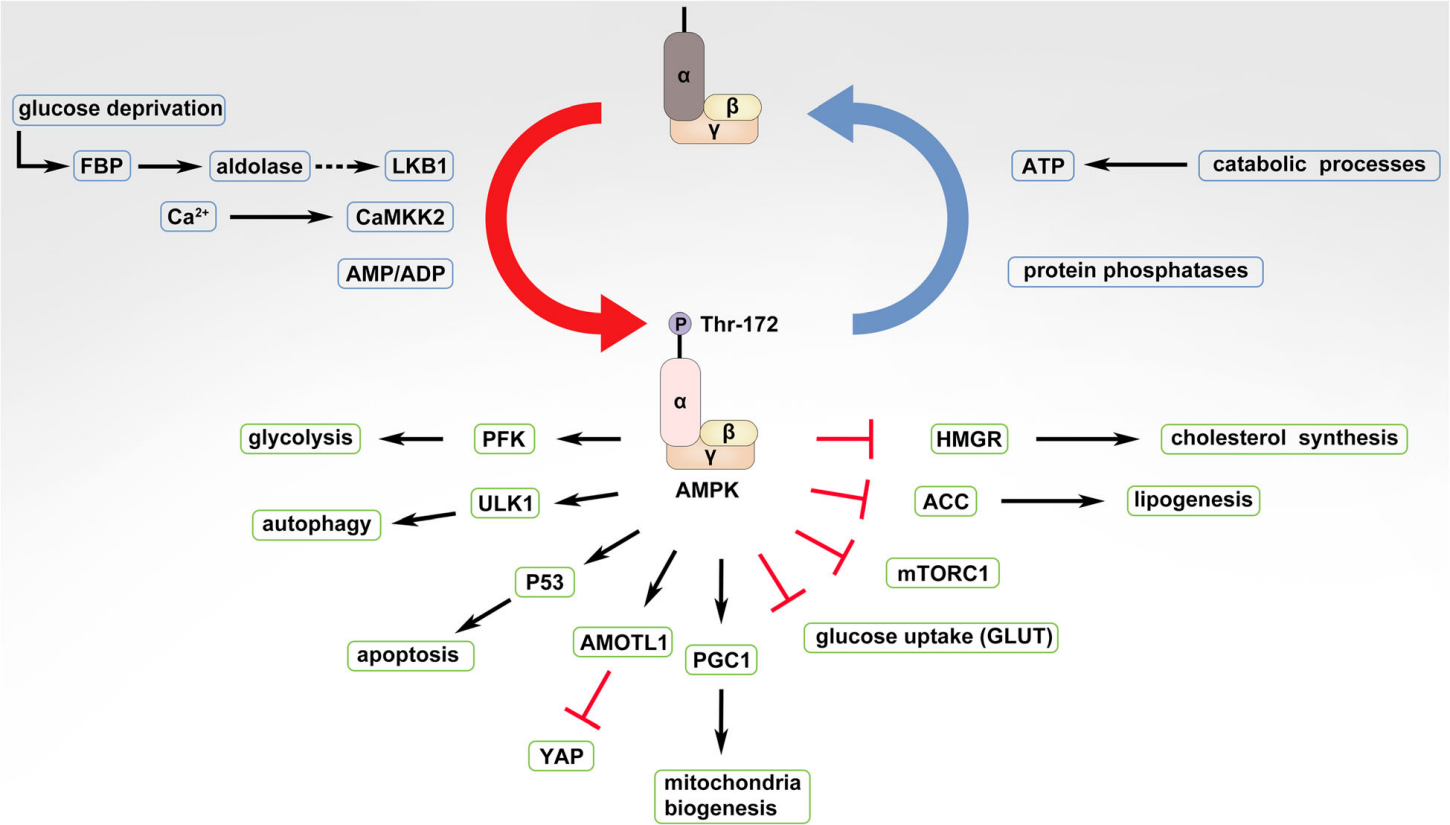
AMPK signaling and its downstream effectors. AMPK is activated by liver kinase B1 (LKB1) or calcium/calmodulin-dependent protein kinase kinase 2 (CAMKK2/β) through phosphorylation on Thr172 of α subunit and is inactivated through dephosphorylation of this site by protein phosphatases in response to changes of cellular AMP:ADP:ATP ratio. Downstream effectors activated by AMPK are indicated as arrows, and those inhibited by AMPK are shown as bar-headed lines

AMPK (AMP-activated protein kinase) is an energy sensor that monitors the AMP:ADP:ATP ratio in eukaryotic cells. This atypical protein kinase was firstly discovered as a contaminant during the purification of acetyl-CoA carboxylase (ACC), a well-studied substrate of AMPK for fatty acid (FA) synthesis nowadays [134–136] (Fig. 2). However, the phosphorylation of ACC by AMPK in response to the high AMP/ATP ratio had not been revealed until a decade later [137], and the enzyme was thus named as AMPK thereafter [138] (Fig. 2). Biochemical studies have shown that AMPK consists of three subunits including the catalytic α subunit and the regulatory β and γ subunits [139–148] (Fig. 2). In mammals, AMPK subunits are encoded as several isoforms (α1, α2; β1, β2; γ1, γ2, γ3), which are preferentially expressed in specific tissues or organisms [145, 149, 150]. For instance, the α2 subunit associates only with β1 in type I muscle fibers, while it binds to both β1 and β2 in type II muscle fibers [150, 151]. Also, the liver formulation of AMPK subunits differs among species as that α1β2γ1 is dominant in human whereas α1β1γ1 and α2β1γ1 in dog and rat, respectively [152]. Although an isoform replacement of AMPK subunits may not extensively affect the basal activity of AMPK as adaptive responses such as exercise do [153], it alters AMPK’s subcellular locations and sensitivity as well as interactions with other signaling pathways [147]. The organism/tissue/stage-specific selectivity of subunit isoforms complicates AMPK’s regulation.

As a key sensor of cellular energy stress, the activity of AMPK is predominantly regulated by cellular AMP/ADP/ATP that competitively binds to the γ subunit of AMPK and thus promotes or inhibits the phosphorylation of Thr172 on α subunit by the tumor suppressor liver kinase B1 (LKB1) or the dephosphorylation of this site by phosphatases [154, 155] (Fig. 2). Besides adenine nucleotides, intracellular calcium ions activate AMPK through calcium/calmodulin-dependent protein kinase kinase 2 (CAMKK2, also called CAMKKβ) [156–158] (Fig. 2), which acts downstream of the hormone-activated receptors such as muscarinic receptors and ghrelin receptor on endothelial cells or neuron cells [159–162]. On the other hand, AMPK can be inhibited by a metabolite of glucose, fructose 1,6-bisphosphate (FBP), which binds to the aldolase and prevents the interaction of AMPK with LKB1 in glucose-rich environments [163] (Fig. 2). Active AMPK has more than 100 downstream substrates that regulate the metabolism of lipids, cholesterol, carbohydrates, and amino acids.

Active AMPK promotes the oxidation of fatty acids and inhibits the synthesis of fatty acids and cholesterol, which involves largely in acetyl-CoA. AMPK phosphorylates and inhibits HMG-CoA reductase (HMGR) that requires acetyl-CoA in its reduction reaction [138, 164, 165] (Fig. 2). Also, AMPK phosphorylates ACC that converts acetyl-CoA to malonyl-CoA and therefore slows down the de novo fatty acid (FA) synthesis and increases the FA oxidation [166] (Fig. 2). Alternatively, AMPK regulates the lipid metabolism through altering the mitochondria structure and function. In the mitochondria, AMPK phosphorylates A-kinase anchoring protein 1 (AKAP1), a key scaffold protein for protein kinase A (PKA), and facilitates the phosphorylation of a mitochondria fusion factor, dynamin-related protein 1 (DRP1) by PKA, which promotes mitochondrial fusion and oxidative phosphorylation [167]. Moreover, AMPK accelerates the mitochondria biogenesis likely through phosphorylating and activating the transcriptional activator, proliferator-activated receptor gamma coactivator 1-alpha (PGC1α) [168, 169] (Fig. 2). However, upon energy stress, AMPK plays an opposite role in mitochondria biology. Under this condition, AMPK is essential for the fragmentation of mitochondria. AMPK phosphorylates mitochondrial fission factor (MFF) on Ser129 and thereby facilitates the translocation of DRP1 from cytosol to mitochondria membrane in energy stress-driven mitochondria fission [170, 171]. Then, AMPK promotes the clearance of damaged mitochondria through autophagy. In this process, AMPK binds directly to and phosphorylates the unc-51-like autophagy activating kinase 1 (ULK1), Autophagy-related gene 9 (ATG9), and Beclin 1, which triggers the autophagosome formation [172–175] (Fig. 2).

Active AMPK directly regulates the carbohydrate metabolism or indirectly through altering the fatty acid metabolism as described above. Activation of AMPK stimulates the expression and plasma membrane translocation of solute carrier family member (GLUT) proteins and thereby facilitates glucose import [152, 176–181] (Fig. 2). Intracellularly, AMPK phosphorylates and activates 6-phosphofructo-2-kinase (PFK2) that is responsible for the synthesis of fructose 2,6-bisphosphate, a potent stimulator of glycolysis, and thus accelerates glycolysis [182] (Fig. 2). Furthermore, AMPK appears to phosphorylate and inhibit glycogen synthase in the liver, which dampens glycogen synthesis and thus indirectly enhances glycolysis [183].

Active AMPK maintains cellular amino acid homeostasis mainly by controlling the activity of mammalian target of rapamycin complex 1 (mTORC1). The mTORC1 is a central sensor of cellular amino acids that samples amino acids in both cytosol and lysosome [184, 185]. Upon activation by amino acids, mTORC1 stimulates protein synthesis by phosphorylating ribosomal protein S6 kinase B1 (S6K) and eukaryotic translation initiation factor 4E binding protein 1 (4E-BP1), which enhances the consumption of cellular amino acids. Moreover, active mTORC1 blocks cellular autophagy by phosphorylating ULK1 and impairs the recycling of amino acids [186]. Both effects of mTORC1 lead to a remarkable drop of cellular amino acid reservoir. Active AMPK has been shown to inhibit the activity of mTORC1 direct and indirectly upon energy stress, which limits the expenditure of amino acids. Alternatively, active AMPK can restrict protein synthesis by phosphorylating and thereby inhibiting eukaryotic translation elongation factor 2 (eEF2) kinase, a key regulator of protein synthesis [187]. To restore cellular amino acid reservoir, active AMPK stimulates cellular autophagy as discussed above, which degrades surplus or dysfunctional proteins into amino acids [186]. In addition, it is worth noted that cellular amino acids can affect the activity of AMPK reversely. Dependent on conditions/contexts, either amino acids may inhibit or stimulate the activity of AMPK though underlying molecular mechanisms remain ambiguous [188–190].

### AMPK signaling in cancer biology

It is well known that AMPK is a putative substrate of tumor suppressor, LKB1 [154, 155, 191] (Fig. 2). Therefore, AMPK has been generally considered as a key effector that mediates the tumor-suppressive function of LKB1. Indeed, a genetic ablation of the AMPK α subunit in mice accelerates Myc-driven lymphomagenesis through facilitating a metabolic shift to aerobic glycolysis [192]. Simultaneously, AMPK inhibitors (AMPKi) promote epithelial-to-mesenchymal transition (EMT) in breast and prostate cancers [193]. These studies validate AMPK as a tumor suppressor under certain circumstances. Further mechanistic studies have demonstrated that AMPK prevents cancers through phosphorylating multiple targets that play indispensable roles on different layers of disease progression. AMPK phosphorylates angiomotin like 1 (AMOTL1), an adaptor protein in the Hippo-Yap pathway, and thus blocks Yes1 associated transcriptional regulator (YAP) activity, which impairs cancer cells’ proliferation and survival [194]. AMPK also phosphorylates TSC complex subunit 2 (TSC2) and regulatory associated protein of MTOR complex 1 (Raptor) and thereby inactivates mTORC1 [195, 196], which in turn elevates cellular autophagy activity and inhibits cancer initiation. To bypass this inhibitory effect, cancer cells can activate the MAGE family member A 3/6 (MAGEA3/6)-tripartite motif containing 28 (TRIM28) ubiquitin ligase complex that targets the AMPK α subunit for degradation and thus re-activates mTORC1 to restrict cellular autophagy [197]. Moreover, AMPK is able to phosphorylate enhancer of zeste 2 polycomb repressive complex 2 subunit (EZH2) and thereby disrupts the polycomb repressive complex 2 (PRC2), which relieves the epigenetic silence of tumor suppressors in cancers [198]. Alternatively, AMPK phosphorylates and stabilizes another epigenetic master regulator, Tet methylcytosine dioxygenase 2 (TET2), which functions as a putative tumor suppressor to prevent tumorigenesis [199]. Altogether, these findings indicate that AMPK has a pronounced anti-tumor activity as its upstream kinase, LKB1 does.

Although significant studies have shown that AMPK dampens the pathogenesis of cancers, some emerging findings indicate that it may promote disease progression under other circumstances. In T cell acute lymphoblastic leukemia (T-ALL), oncogenic Notch signaling induces a high level of aerobic glycolysis which needs to be restrained by AMPK, and loss of AMPK results in energy stress-driven apoptosis of leukemic cells and slows down disease progression [200]. Similarly, in acute myeloid leukemia (AML), metabolic stress elevates the ROS level and induces DNA damage in leukemia-initiating cells (LICs), and AMPK confers metabolic stress resistance to LICs [201]. AMPK knockout or pharmaceutical inhibition under metabolic stress kills LICs and inhibits leukemogenesis. Moreover, AMPK plays a determinant role in maintaining the NADPH homeostasis in cancer cells upon energy stress, which is critical for cancer cell survival [202]. Depletion of the AMPK α subunit or its upstream kinase, LKB1 makes cancer cells susceptible to death upon energy stress, such as glucose limitations, anchorage-independent growth, and solid tumor formation in vivo. In Kras^G12D^-driven non-small cell lung cancer, the failure of AMPK activation by virtue of LKB1 mutation sensitizes cancer cells for phenformin-induced metabolic stress, further supporting that AMPK adapts cancer cells for metabolic stress [203]. Alternatively, a synthetic lethal screening has revealed that AMPK activation by AMPK-related kinase 5 (ARK5) is essential for Myc-driven cancer progression [204]. Consistent with this finding, AMPK has been shown to promote survival of Myc-positive melanoma cells with N-Ras mutation by restraining oxidative stress [205]. In addition, AMPK sustains the activation of oncogenic protein kinase B (AKT) signaling upon stress or epidermal growth factor receptor (EGFR) engagement in breast cancers [206]. Besides these direct effects on cancer cells, AMPK may promote cancer progression by altering the cancer microenvironment. AMPK signaling has been shown to intrinsically promote the immunoregulatory activity of myeloid-derived suppressor cells (MDSC), which dysfunctions T cells in cancer tissue [207]. All these findings indicate that AMPK can significantly contribute to the disease progression of variable cancers via distinct manners.

Unlike LKB1, which is frequently mutated or deleted in cancer genomes [208–210], AMPK has nearly no mutations, and on the contrary, is upregulated in some types of cancers such as glioblastoma [211], suggesting that it may play a paradoxical role in carcinogenesis. Dependent on origins of cancers, driver mutations, developmental stages, and external conditions, AMPK may dampen or promote the disease progression of cancers, and uncovering underlying mechanisms would propel cancer therapy development by targeting this signaling pathway.

## The crosstalk between MAPK and AMPK signalings

**Fig. 3:**
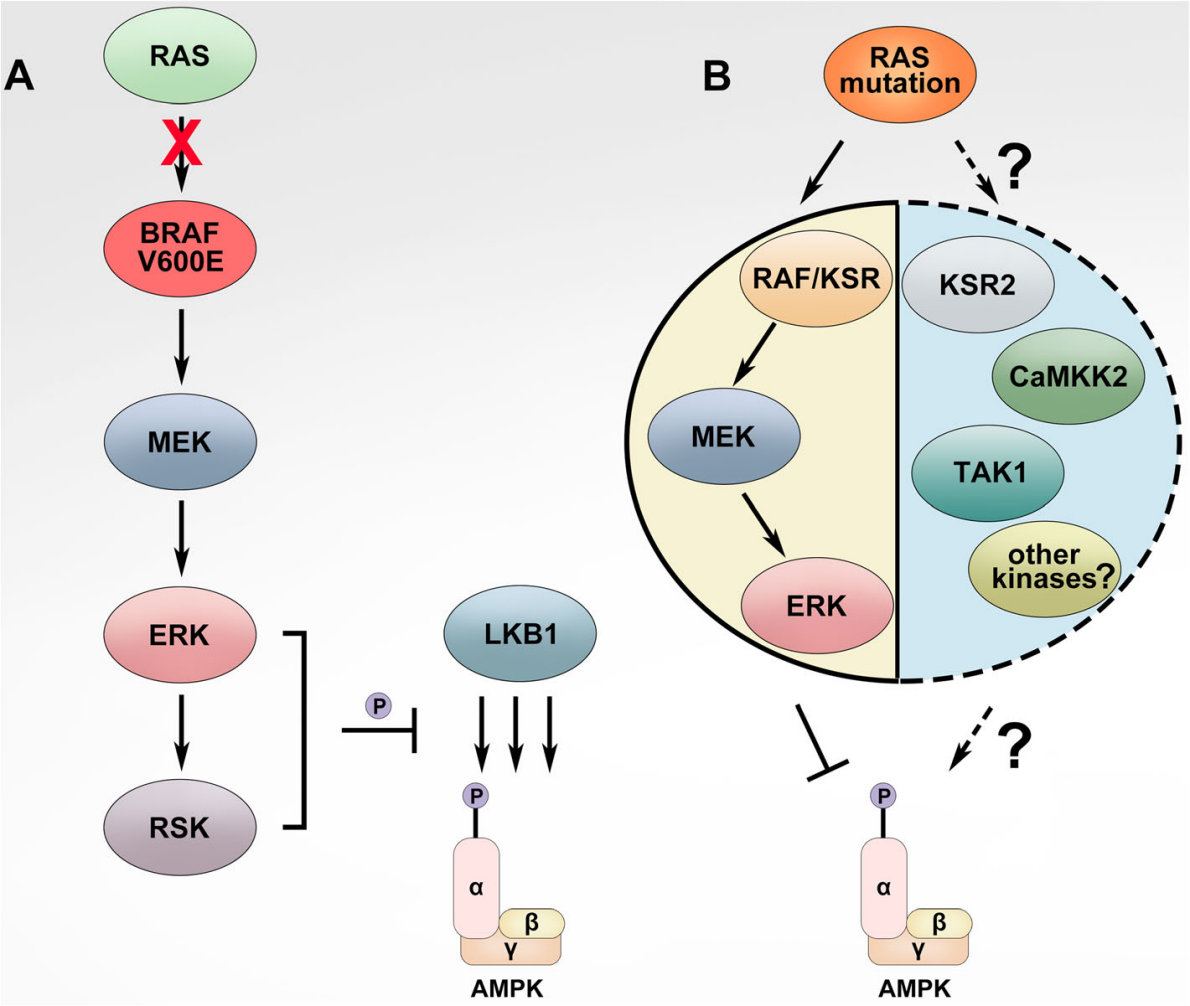
AMPK signaling is inhibited by hyperactive Ras/RAF/MEK/ERK (MAPK) signaling in cancers. **a** In BRAF(V600E)-harboring cancers, hyperactive ERKs and downstream RSKs phosphorylate LKB1 on Ser325 and Ser428 sites, which inactivates LKB1 and thus blocks the activation of AMPK by LKB1. **b** In Ras-mutated cancers, the activity of AMPK is partially inhibited likely by hyperactive MAPK signaling, though the underlying molecular mechanism remains ambiguous. However, this moderate AMPK activity is indispensable for disease progression in Ras-mutated cancers

As described above, the MAPK signaling controls cellular proliferation, differentiation, and survival, whereas the AMPK signaling regulates cellular metabolism. However, many studies have indicated that these two distinct signalings have profound and complicated interplays in both physiological and pathological processes. In quiescent cells, the AMPK signaling maintains energy homeostasis by switching on catabolic pathways that generate ATP, while switching off anabolic pathways that are required for cell growth [142, 146, 176–183, 204, 212–215]. Upon mitogen stimulation, the MAPK signaling is turned on and drives cellular proliferation/differentiation, which needs cells shifting their metabolic program from catabolic to anabolic for biomass synthesis [216, 217]. To achieve this, the MAPK signaling activates transcription factors such as Myc and Hypoxia inducible factor 1 subunit alpha (HIF-1α), which control the expression of glycolytic enzymes and promote aerobic glycolysis [218–221]. Furthermore, the MAPK signaling directly regulates AMPK signaling and thus constrains the AMPK signaling-driven oxidative phosphorylation of biomaterials [167, 222]. These interplays frequently occur with marginal coordination when cells response to different stimuli such as oncogenesis and cell stress. Recent studies have revealed that the MAPK signaling regulates AMPK signaling on different layers under distinct circumstances. Firstly, ERK and ribosomal protein S6 kinase A (RSK), two downstream kinases of MAPK signaling, have been shown to phosphorylate and inhibit the upstream activator of AMPK, LKB1, and thereby block the activation of AMPK by LKB1 in BRAF(V600E)-driven melanoma [223] (Fig. 3a). Secondly, ERK likely phosphorylates the α subunit of AMPK directly on negative regulatory sites Ser485/491 and impairs its catalytic activity, which is essential for C-C motif chemokine receptor 7 (CCR7)-dependent survival of mature dendritic cells [224]. Thirdly, KSR, one of the key components of MAPK module, has been shown to interact with all AMPK subunits and regulate the AMPK-dependent energy expenditure [225, 226] (Fig. 3b). In addition, the MAPK signaling controls the subcellular localization of AMPK and thus alters its function under cell stress [227]. All these findings suggest that AMPK could function as a downstream effector of MAPK signaling.

**Fig. 4:**
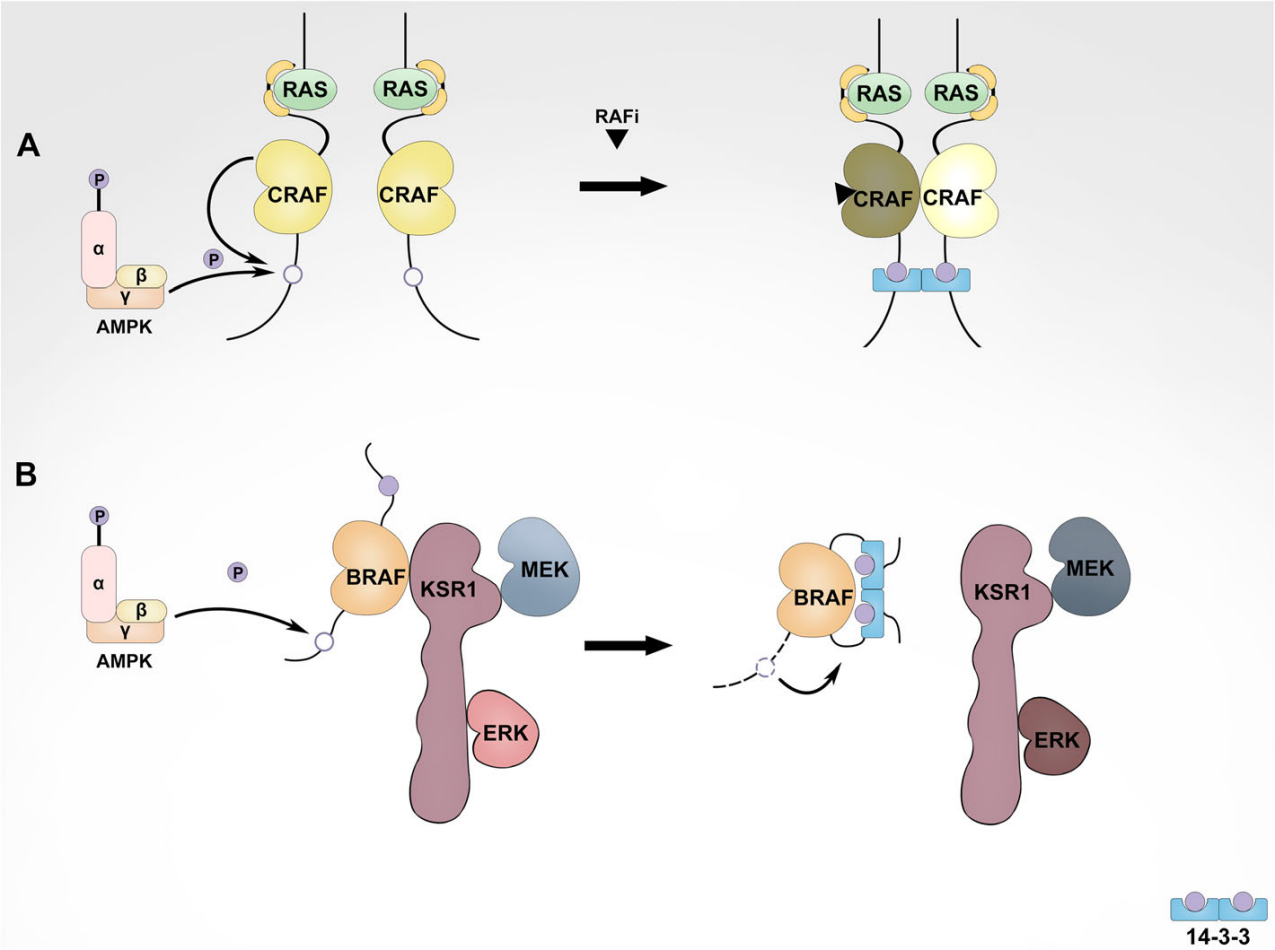
AMPK regulates differentially hyperactive Ras/RAF/MEK/ERK (MAPK) signaling in Ras- versus BRAF(V600E)-mutated cancers. **a** In Ras-mutated cancers, the C-terminal 14-3-3 binding site of CRAF is phosphorylated by AMPK, which facilitates CRAF dimerization through improving the association of CRAF dimer with 14-3-3 dimer and thus elevates the activity of CRAF, particularly upon RAF inhibitor treatment or metabolic stress. Under these conditions, CRAF forms homodimers with itself or heterodimers with KSR or BRAF. **b** In BRAF(V600E)-harboring cancers, AMPK phosphorylates the C-terminal 14-3-3 binding site of BRAF(V600E), which prevents BRAF(V600E) dimerization with KSR through enhancing the association of a single BRAF(V600E) molecule with 14-3-3 dimer and thus blocks the activity of BRAF(V600E) upon metabolic stress

The interplays between MAPK and AMPK signalings are binary, and the AMPK signaling can regulate MAPK signaling reversely. Conclusive evidence shows that AMPK can directly phosphorylate the RAF/KSR family kinases, the pivotal components of MAPK module, and alter their activities under variable conditions. It is well established that the hetero-/homo-dimerization of RAF/KSR family kinases plays a determinant role in the activation of MAPK signaling, which requires the association of 14-3-3, a dimeric scaffold protein with their carboxyl-terminus [228, 229]. Mechanistic studies have revealed that a 14-3-3 dimer associates with the C-terminus of two individual RAF/KSR molecules and facilitates their dimerization and subsequent activation [25, 230, 231] (Fig. 4a). Since RAF/KSR family kinases have the other conserved 14-3-3 binding site at the N-terminus, however, if a 14-3-3 dimer binds to the N- and C-terminus of a single RAF/KSR intramolecularly, it will stabilize RAF/KSR in an autoinhibitory conformation and thus prevent the dimerization-driven activation of kinases [38, 77, 232] (Fig. 4b). AMPK has been shown to phosphorylate the C-terminal 14-3-3 binding site of RAF/KSR family kinases and promote the intra- or inter-molecular 14-3-3 associations with these kinases respectively [233, 234] (Fig. 4). Among RAF/KSR family kinases, CRAF is the first member that has been shown being phosphorylated by AMPK on its C-terminal 14-3-3 binding site [234]. AMPKi by pharmaceutical inhibitors abolishes the dimer-dependent paradoxical activation of MAPK signaling driven by the RAF inhibitors in Ras-mutated cancers, suggesting that AMPK-mediated phosphorylation of the C-terminal 14-3-3 binding site on CRAF promotes the intermolecular association of 14-3-3 dimers with CRAF homo- or hetero-dimers [231] (Fig. 4a). This molecular mechanism may also be responsible for the hyperactive MAPK signaling induced by metabolic stress in Ras-mutated melanoma. Upon metabolic perturbations, AMPK is activated in this type of melanoma cells and promotes KSR/CRAF heterodimerization likely through altering 14-3-3 binding manners, which leads to a highly activated MAPK signaling [230]. Besides CRAF and KSR, the association of BRAF with 14-3-3 is also regulated by AMPK-mediated phosphorylation. In BRAF(V600E)-harboring melanoma, metabolic stress-activated AMPK phosphorylates the C-terminal 14-3-3 binding site of BRAF and promotes the intramolecular association of a single BRAF molecule with a 14-3-3 dimer [233], which breaks the BRAF/KSR heterodimer and thus inhibits MAPK signaling [230], although whether active AMPK phosphorylates the N-terminal 14-3-3 binding site of BRAF under this condition needs further investigation (Fig. 4b). Consistent with these findings, AMPK activators have been shown to inhibit the proliferation of BRAF(V600E)-harboring melanoma and enhance the therapeutic efficacy of BRAF inhibitors on this type of melanoma [235, 236]. Over all, the distinct regulations of RAF/KSR family kinases by AMPK lead to completely different outputs of MAPK signaling, which determine cell fates under variable conditions.

The interplays between MAPK and AMPK signalings also alter cellular autophagy, particularly that of cancer cells. Cancer cells with Ras/RAF mutations have much higher activity of autophagy [237–240], which significantly contributes to disease progression [238, 240–246], although how autophagy is upregulated in these cancer cells remains unknown. Elevated autophagy in Ras/RAF-mutated cancer cells preserves mitochondrial and glycolytic functions by recycling dysfunctional mitochondria [247, 248]. Disruption of autophagy by depleting Atg7 or Atg5 induces cellular senescence and reduces cancer burden in these diseases [238, 240–246]. The critical role of autophagy in K-ras-driven cancers is further confirmed by a synthetic lethal screening for factors that support K-ras addiction, which identified Atg7 and RAF kinases as a minimal oncoeffector combination that best discriminates K-ras cancer cells from normal cells [249]. It is well known that AMPK is a prominent regulator of autophagy in spite of its key role as an energy sensor, which drives cellular autophagy machinery via the LKB1/AMPK/ULK1 axis [250–252]. Since LKB1 is inhibited by hyperactive MAPK signaling, this signal axis should not be responsible for elevated activity of autophagy in Ras/RAF-driven cancers. However, it provides cancer cells a protective strategy for adapting themselves to MAPK inhibition [248, 253, 254]. Indeed, MAPK inhibition (MAPKi) by RAF/MEK/ERK inhibitors in Ras/RAF-mutated cancer cells further elevates autophagic flux through AMPK, which restores cellular metabolic hemostasis and leads to tolerance towards MAPKi.

## Combinatorial targeting of MAPK and AMPK signalings to treat Ras/RAF-mutated cancers

**Fig. 5:**
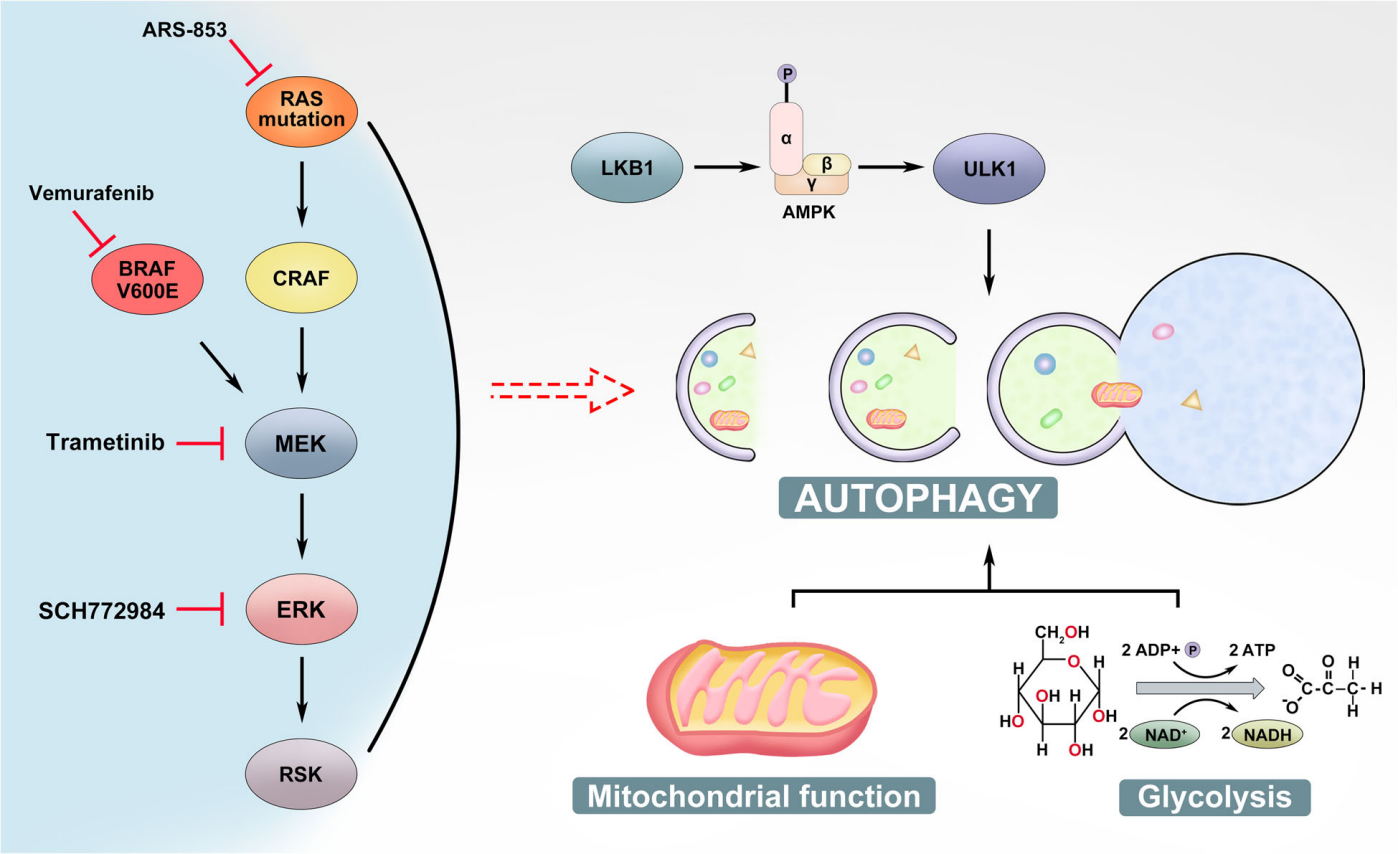
Combinatorial targeting of hyperactive Ras/RAF/MEK/ERK (MAPK) signaling and AMPK-mediated autophagy to treat Ras/RAF-mutated cancers. Blocking hyperactive Ras/RAF/MEK/ERK (MAPK) signaling by MAPK inhibitors in Ras/RAF-mutated cancer cells elevates autophagic flux through relieving LKB1/AMPK/ULK1 axis and inhibiting glycolysis and mitochondrial functions, which leads to drug tolerance and/or acquired resistance. Combinatorial inhibition of both hyperactive MAPK signaling and autophagy remarkably improves therapeutic efficacy of drugs against these cancers

Hyperactive MAPK signaling is responsible for a large portion of cancers, and genetic alterations that aberrantly activate this pathway mainly occur on receptor tyrosine kinases (RTKs), Ras small GTPases, and BRAF [63]. In current cancer therapies, hyperactive RTKs can be effectively targeted by tyrosine kinase inhibitors (TKIs) or neutralizing antibodies [255–263], while there are no drugs that are able to specifically target most Ras mutants [264]. To treat Ras/BRAF-mutated cancers, RAF/MEK inhibitors such as vemurafenib, dabrafenib, encorafenib, trametinib, cobimetinib, and binimetinib have been developed and applied to disease management [99–101, 265]. These inhibitors have exhibited a promising efficacy towards most BRAF-mutated cancers [101, 108–116] (Fig. 5). In contrast, Ras-mutated cancers are intrinsically resistant to these drugs, which do not inhibit but paradoxically activate the MAPK signaling through promoting RAF family kinases’ dimerization [266]. Furthermore, even BRAF-mutated cancers develop adaptive resistance to these drugs after 6~10 months treatment by either activating Ras or alternatively splicing BRAF mutant [266]. Therefore, for most cases, once cancer cells possess high Ras activity, these drugs lose their efficacy as a monotherapy. To improve the efficacy of MAPKi against Ras/RAF-mutated cancers, emerging evidence indicates that disruption of MAPK signaling complex, particularly dimerization of RAF family kinases, and/or synergistic targeting of synthetic lethality of MAPK signaling should be two feasible strategies [267–272], both of which are involved in AMPK signaling.

It has been shown that the components of MAPK signaling form a super complex in cancer cells with active Ras [273, 274], which leads to resistance towards MAPKi. Assembly of this complex involves in RAF/RAF (or KSR), RAF (or KSR)/MEK, MEK/MEK, as well as RAF (or KSR)/14-3-3 interactions, and disruption of these interactions contributes to an effective inhibition of MAPK signaling. As discussed above, AMPK directly regulates RAF (or KSR)/14-3-3 interaction by phosphorylating the 14-3-3 binding sites on RAF (or KSR) and thus facilitates or impairs RAF/RAF (or KSR) dimerization. In Ras-mutated cancer cells or RAFi-resistant cancer cells with active Ras, CRAF is the key isoform of RAF family kinases responsible for disease progression and drug resistance [27, 32, 275–278], whose phosphorylation on the C-terminal 14-3-3 binding site by AMPK plays a determinant role in the paradoxical effect of RAF inhibitors, and AMPKi sensitizes these cancer cells to RAF inhibitors both in vitro and in vivo [231] (Wang & Hu, unpublished data) (Fig. 4b). Although the components of MAPK signaling do not assemble a super complex in BRAF-mutated cancer cells, constitutively active BRAF mutant still functions as homo- or hetero-dimers (BRAF/BRAF or BRAF/KSR) that can be disrupted by AMPK-driven phosphorylation of both N- and C-terminal 14-3-3 binding sites [230, 231, 233]. In this type of cancers, AMPK activators have been shown to significantly enhance the therapeutic efficacy of RAF inhibitors [235, 236]. Taken together, altering RAF/KSR dimerization by using either AMPK inhibitors or activators may remarkably improve the targeted therapies of Ras/RAF-mutated cancers with RAF inhibitors.

Since most oncogenic Ras mutants are undruggable, efforts for developing effective approaches against Ras-mutated cancers have been switched to identify and target synthetic lethal vulnerabilities of Ras mutants over decades, which led to the discovery of some putative factors essential for in vitro growth of Ras-mutated cancer cells [267, 279]. Unfortunately, most factors except those regulating cellular autophagy exhibit little-to-no therapeutic values for treating Ras-mutated cancers in vivo so far. As we know, Ras/RAF-mutated cancer cells have a high basal activity of autophagy though hyperactive MAPK signaling inhibits the LKB1-AMPK-ULK1 signaling axis, which is critical for maintaining cellular metabolic homeostasis. MAPKi relives the LKB1-AMPK-ULK1 axis and thus further elevates autophagic flux in Ras/RAF-mutated cancer cells, which adapts Ras-mutated cancer cells to MAPKi [25, 254], or promotes drug tolerance and subsequent resistance of RAF-mutated cancer cells [253] (Fig. 5). Pharmaceutical blocking of AMPK by compound C has been shown to remarkably reduce the RAFi-resistant clones arising from BRAF-mutated melanoma [231]. Furthermore, combinations of autophagy inhibitors with RAF/MEK/ERK inhibitors (chloroquine plus vemurafenib, hydroxychloroquine plus trametinib, or chloroquine plus SCH772984) can effectively block the growth of K-ras-mutated pancreatic ductal adenocarcinoma, N-ras-mutated melamona, as well as BRAF-mutated colorectal cancer and melanoma in vivo [248, 253, 254, 280] (Fig. 5). However, it has to be noted that although both AMPK inhibitors and activators may synergistically enhance the therapeutic efficacy of RAF inhibitors against BRAF-mutated cancers, molecular mechanisms underlying these phenomena are completely different.

Combinatorial inhibition of both MAPK and AMPK signalings has shown promising potentials for treating Ras/RAF-mutated cancers. To target MAPK signaling, the first-generation RAF/MEK/ERK inhibitors have been developed and applied to clinic treatment, and the second-generation drugs that can inhibit RAF mutants with elevated dimer affinity or have much less paradoxical effect have also undergone clinical trials [102, 121, 281, 282]. In contrast, the drug development of AMPK-specific activators and inhibitors lags far behind the needs for treating cancers, though some AMPK activators such as O304 are undergoing clinical trials for other diseases [148]. Currently, only two non-specific AMPK activators, phenformin and metformin, that have been approved for treating type II diabetes are undergoing clinic evaluations as combinatorial agents for treating BRAF-mutated melanoma together with vemurafenib or dabrafenib/trametinib. As to AMPK-specific inhibitors, only compound C has been tested in animal models at present [283]. Although a combination of compound C with RAF inhibitor, vemurafenib can effectively inhibit the growth of Ras-mutated cancer cells in vitro [231], and its therapeutic efficacy/benefit needs further investigations by using preclinical animal models and through clinical trials. Overall, these unmet needs for AMPK-specific activators and inhibitors in targeted cancer therapies appeal to accelerate their pharmaceutical development.

## Conclusions and perspectives

Recent studies have provided compelling evidence that interplays between MAPK and AMPK signalings play a critical role in cell physiology and have important implications in disease treatment, particularly for cancer. Combinatory targeting both MAPK and AMPK signalings represents for a promising therapeutic intervention. However, although the framework by which these two signalings interact with each other has been illustrated, the precise molecular basis and their impacts on cancer therapies remain largely unresolved. For instance, how the AMPK signaling differentially regulates the dimerization of different RAF isoforms (BRAF versus CRAF) and thus distinctly alters the outputs of MAPK signaling in Ras- versus RAF-mutated cancers is unclear. Besides elevating autophagic flux, does the AMPK signaling plays other roles in the MAPKi-resistance of Ras/RAF-mutated cancers? Addressing these questions would deepen our understanding of MAPK/AMPK interplays and help us develop better combinatorial therapies for cancers and other diseases. In addition, developing AMPK-specific activators/inhibitors would be an attractive research topic for both academy and pharmaceutical industry in the next years given their absence and unmet needs in clinic treatment.

## Data Availability

Not applicable.

## Abbreviations

MAPK: Mitogen-activated protein kinase
AMPK: AMP-activated protein kinase
Ras: Ras proto-oncogene
GTPase; RAF: Raf proto-oncogene, serine/threonine kinase
MEK: Mitogen-activated protein kinase kinase
ERK: Mitogen-activated protein kinase
KSR: Kinase suppressor of Ras
CR: Conserved region
RBD: RAS-binding domain
NTA: N-terminal acidic motif
PI3K: Phosphoinositide 3-kinase
AKT: Protein kinase B
mTORC: Mammalian target of rapamycin complex
RTKs: Receptor tyrosine kinases
GEFs: GTP/GDP exchange factors
GAPs: GTPase activating proteins
SHP2: Src homology region 2 domain-containing phosphatase-2
ACC: Acetyl-CoA carboxylase
FA: Fatty acid
LKB1: Liver kinase B1
CAMKK2: Calcium/calmodulin-dependent protein kinase kinase 2
FBP: Fructose 1,6-bisphosphate
HMGR: HMG-CoA reductase
AKAP1: A-kinase anchoring protein 1
PKA: Protein kinase A
DRP1: Dynamin-related protein 1
PGC1α: Proliferator-activated receptor gamma coactivator 1-alpha
MFF: Mitochondrial fission factor
ULK1: Unc-51-like autophagy activating kinase 1
ATG: Autophagy-related gene
GLUT: Solute carrier family 2 member
PFK2: 6-Phosphofructo-2-kinase
S6K: Ribosomal protein S6 kinase B1
4E-BP1: Eukaryotic translation initiation factor 4E binding protein 1
eEF2: Eukaryotic translation elongation factor 2
EMT: Epithelial-to-mesenchymal transition
AMOTL1: Angiomotin like 1
YAP: Yes1 associated transcriptional regulator
TSC2: TSC complex subunit 2
Raptor: Regulatory associated protein of MTOR complex 1
MAGEA: MAGE family member A
TRIM28: Tripartite motif containing 28
EZH2: Enhancer of zeste 2 polycomb repressive complex 2 subunit
PRC2: Polycomb repressive complex 2
TET2: Tet methylcytosine dioxygenase 2
T-ALL: T cell acute lymphoblastic leukemia
AML: Acute myeloid leukemia
LICs: Leukemia-initiating cells
ARK5: AMPK-related kinase 5
EGFR: Epidermal growth factor receptor
MDSC: Myeloid-derived suppressor cells
HIF-1α: Hypoxia inducible factor 1 subunit alpha
RSK: Ribosomal protein S6 kinase A
CCR7: C-C motif chemokine receptor 7
